# Child Sexual Abuse in Nigeria: A Systematic Review

**DOI:** 10.1177/15248380241254077

**Published:** 2024-05-29

**Authors:** Moninuola Ifayomi, Parveen Ali, Katie Ellis

**Affiliations:** 1 University of Sheffield, UK; 2 Sheffield University Interpersonal Violence Research Group, UK

**Keywords:** child sexual abuse, child abuse, sexual abuse, victims, perpetrators, Nigeria and systematic review.

## Abstract

Child sexual abuse (CSA) is a major social and public health issue that creates short- and long-lasting impacts on victims, families, and society. While global researchers have considered the topic of CSA since the 19th century, the Nigerian context has been largely ignored. Yet, without sufficient evidence and understanding, making changes to practices and policies becomes almost impossible. The review aimed to gain insights into the nature and extent of CSA and identify areas for improvement in practice and research in Nigeria. This article presents the findings of a systematic review of 31 empirical articles related to CSA in Nigeria. Using key search terms along Boolean operators and truncation, PubMed, PsycINFO, CINAHL, ASSIA, PILOTS, African Journals Online, and Google Scholar were searched. A total of 1,325 studies were found, and 31 empirical studies, including 20 quantitative, 9 qualitative, and 2 mixed methods studies, were included. The review findings reveal the discourse on CSA and delve into various aspects such as its prevalence, manifestation patterns, root causes, management, and consequential impact on victims and societal domains. The gaps in the existing literature are identified and explored to identify areas for improvement in victim services, societal awareness, and healthcare practices and relevant policies. The sociocultural norms not only heightened children’s vulnerability to sexual abuse but also posed significant barriers to them disclosing such abuse. Survivors of CSA often receive inadequate care, indicating a pressing need for improvements in this area. Implications for research, policy, and conclusion were discussed.

## Introduction

Child sexual abuse (CSA) occurs when a child is involved in sexual activity without understanding or being able to give informed consent or when the activity violates laws or social norms (United Nations International Children’s Emergency Funds, 2015; World Health Organization, 2006; 2014). CSA can be physical or non-physical, penetrative or non-penetrative, and has serious short-term and long-term effects on victims. According to the World Health Organization, one in four children globally experiences some form of sexual abuse. The prevalence of CSA in Nigeria is largely unknown, and estimates range from 2.1% to 77.7% (Audu et al., 2009; Obisesan et al., 1999). More than 31.4% of girls’ first sexual experience in Nigeria was reported to be rape or forced sex (United Nations International Children’s Emergency Funds, 2015). The actual magnitude of CSA remains unknown, with disparities in published statistics across the world (Audu et al., 2009; Pereda et al., 2009; World Health Organization, 2014) as it is highly underreported and underestimated.

The causes of CSA are multifaceted, comprising individual, contextual, and environmental factors that increase a child’s vulnerability (Smallbone et al., 2013). Young children, for example, can be particularly vulnerable to CSA due to their higher level of dependency, inability to protect themselves, and difficulty articulating their experiences or seeking help (Martinello, 2020; Sodipo et al., 2018). In addition, they are sometimes unable to see sexual exploitation when disguised as love, protection, or friendship (Melrose, 2013; Smallbone et al., 2013). In addition, children with a learning or physical disability, children being looked after away from home, and children with interrupted care histories (Coy, 2009; Ellis, 2019) are more at risk of experiencing CSA.

CSA entails short-term and lifelong sequela for the individual, family, and society, especially if left unrecognized or untreated (Hornor, 2010; Warrington et al., 2017). CSA has become a serious challenge in all societies, increasing victims’ risk of developing a wide range of physical and mental problems. The consequences are numerous and pervasive, and they may affect the physical, psychological, emotional, social, moral, educational, and economic wellbeing of victims (Hornor, 2010; Sigurdardottir et al., 2014). Victims of CSA can experience both physical (genital trauma, unplanned pregnancy, and sexually transmitted diseases), psychological (depression, post-traumatic stress disorder, self-blame, distrust, and anger), emotional (fear and anxiety), and behavioral difficulties (mistrust of others and missing from home) (Aborisade & Vaughan 2014; Okeafor et al., 2018; Oteh et al., 2009). Such experiences can also leave victims traumatized by unsavory memories, which can truncate psychosocial development and impede educational careers (Sigurdardottir et al., 2014; Warrington et al., 2017).

The true burden of CSA in Nigeria remains unknown and difficult to determine as most available data are collected through social media and cases presented at hospitals. The research team intends to delve deeper into the perspectives and methods employed by healthcare professionals (HCPs) in their line of work. The culture of silence around CSA has masked the extent of CSA. In addition, limited research has been conducted to aggregate the existing evidence around CSA in Nigeria. The few existing peer-reviewed articles from Nigeria are from clinical cases and do not account for the many cases that never reach a clinic. This leaves out evidence that may be present among children in and out of school, and adolescents from both secondary and tertiary institutions. Therefore, it is important to systematically appraise the few empirical studies available in Nigeria to aggregate the available evidence and understand the nature and extent of CSA in Nigeria. More specifically, the research team was interested in exploring the context in which the HCPs supporting sexually abused children in Nigeria operate.

This review aims to aggregate and critically appraise the quality of the available evidence to identify what was known about CSA in Nigeria, highlight gaps in the existing literature, and develop a theoretical framework to underpin and design subsequent studies. Our specific aim was to systematically review the existing body of knowledge on CSA in Nigeria, the prevalence and pattern of CSA, its causes, determinants, impacts, and HCPs understanding and working practices.

### Methods

#### Eligibility Criteria

A systematic review approach was used to conduct exhaustive searches to identify and review all available peer-reviewed literature on CSA in Nigeria. Studies were considered eligible for review if they were (a) empirical in nature, (b) explored any topics on CSA in Nigeria, (c) published in peer-reviewed scholarly journals, (d) published in English language (which is the official language in Nigeria) (e) published between January 1, 1999, and May 2022. Literature from this period was included for three main reasons: the availability of data, temporal trend in the available evidence, and relevance of evidence. The issue of CSA in Nigeria was often reported in newspapers before 1999, but unfortunately, it was largely overlooked by social systems. However, in 1999, the federal government amended the Constitution of the Federal Republic of Nigeria to outline the country’s fundamental principles, powers, and rights. The amended constitution recognized various forms of child abuse, marking a crucial turning point, which has since led to increased awareness, research, and professional support for CSA victims in Nigeria. Table 1 provides details on the inclusion and exclusion criteria.

**Table 1.** Inclusion and Exclusion Criteria.

Inclusion Criteria	Exclusion Criteria
Focused on Child sexual abuse (CSA) prevalence, pattern and impacts	Studies that did not focus on CSA
Focused on society’s perception of CSA	Studies conducted before January 1st, 1999
Focused on healthcare workers’ knowledge, attitudes, or awareness of CSA	Literature reviews, including either narratives or systematic or meta-analysis reviews and commentaries
Focused on the present CSA intervention in Nigeria, issues and challenges facing healthcare professionals	PhD thesis
Only studies published in the English Language	Not published in English
Between the years January 1st, 1999 to May 2022	Studies conducted before January1st, 1999
Published in peer-reviewed journal	

*Note.* A description of inclusion and exclusion criteria.

### Data Sources

For a comprehensive literature review, the following six databases were systematically searched: PubMed, PSYCINFO, CINAHL, ASSIA, PILOTS, and African Journals Online, as well as Google Scholar, was also used in order not to miss out any studies not indexed. Also, the reference list of all articles included was keenly searched to identify other relevant empirical studies. Boolean operators (AND and OR) and truncation (*) were used alongside some of these keywords: “Child sexual abuse and pattern,” “Prevalence and child sexual abuse,” “Child sexual abuse and causes,” “Child abuse AND Nigeria,” “healthcare professionals AND child sexual abuse,” “Nigeria AND girls AND abuse,” “Nigeria AND sexual child abuse OR sexual exploitation,” “Doctors OR Nurse AND child sexual abuse OR child molestation,” “Nigeria AND issues and challenges AND healthcare providers,” “Barriers AND detecting Child abuse in Nigeria.”

### Study Selection, Quality Appraisal, and Data Extraction

**Figure 1.**
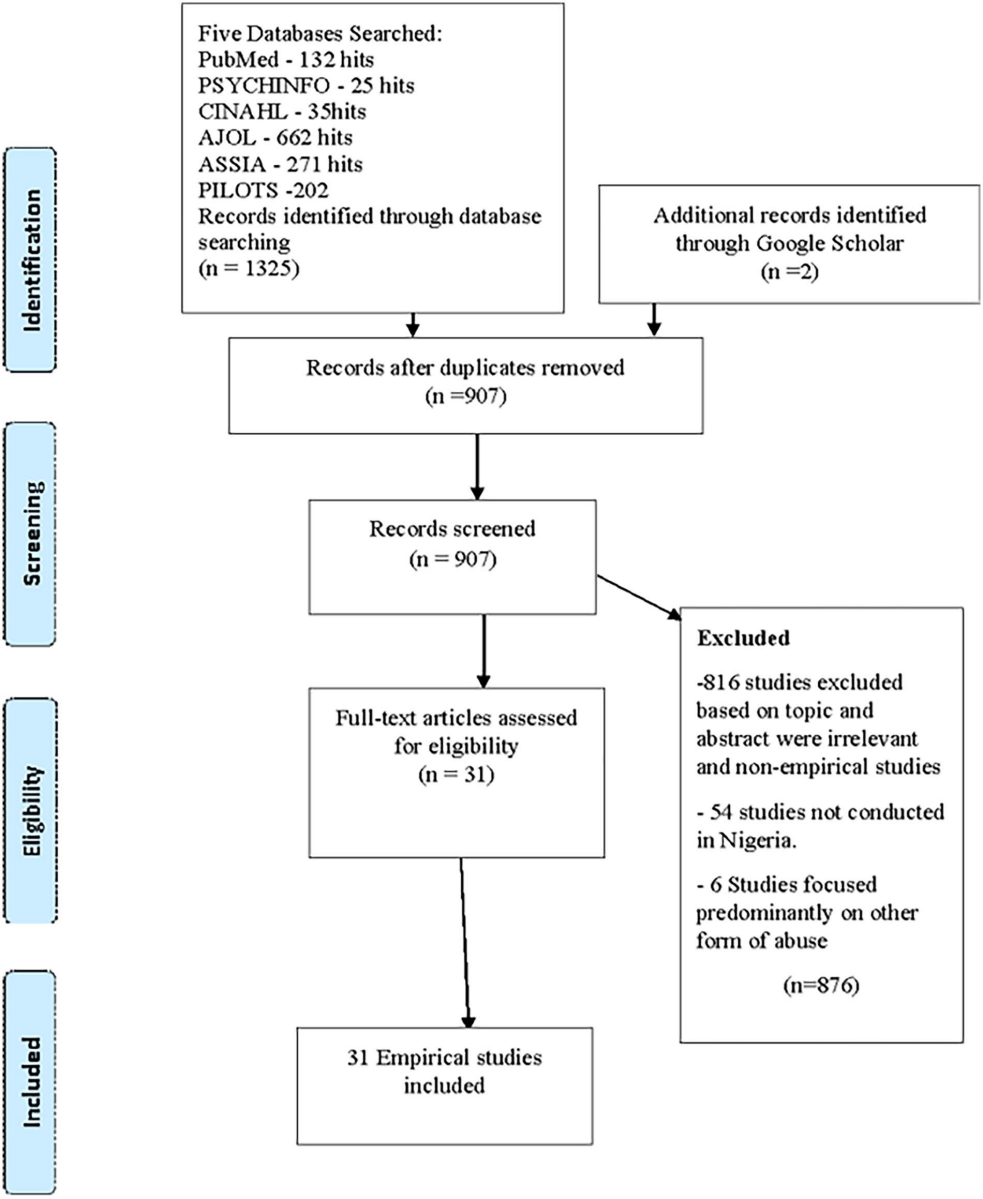
PRISMA flowchart of study selection. *Note.* The process of selecting and reasons for excluding articles for this review.

Following a comprehensive search, a total of 1,325 studies were identified. Of these, 418 were duplicates, and 876 were irrelevant studies (did not meet the inclusion criteria, were not empirical, were not conducted in Nigeria, or did not focus on CSA) were excluded. Review of the titles and abstracts resulted in the selection of 31 studies that met the inclusion criteria. The full text was retrieved for all 31 articles and an independent reviewer read these articles and all peer checked to determine suitability for inclusion. Figure 1 shows the PRISMA (Preferred Reporting Items for Systematic Reviews and Meta-Analyses) flowchart from identification of studies to the inclusion process.

A data extraction form was used to excerpt relevant information, the author names, year of study, aims of the study, design, settings, methodology, sample size, the study’s results and limitations of the studies. Similar concepts were clustered together to present findings. Each study that met the inclusion criteria was quality assessed using the critical appraisal skill program checklists, including the qualitative checklist (for qualitative studies), cohort study checklist (for quantitative studies, and case-control study checklist). The mixed methods appraisal tool (MMAT) Version 2011 was employed for quality evaluation of mixed methods studies. Study results were also analyzed to summarize prevalence of various forms of CSA, patterns of CSA, causes and determinants/predictors, consequences and impact on victims, family and society, society’s awareness and attitude toward CSA and victims and HCPs’ perceptions and practices and available services and identifying issues and challenges. Similar concepts were clustered together to present findings.

## Results: Description of Studies Setting, Population, Design and Methodology

Table 2 summarizes each of the 31 included studies. The majority of these studies were quantitative (*n* = 23) and cross-sectional. Only six studies were qualitative (Aborisade & Vaughan, 2014; Aborisade et al., 2018; Aderinto, 2010; Nwolisa et al., 2016; Olatunya et al., 2013; Oteh et al., 2009), and two were mixed methods (Ogunyemi 2000; Oyekola & Agunbiade 2018). The majority of the studies were conducted in the Southwest (*n* = 19), and only five were carried out in the Southeast. One was conducted in each remaining geographical zone except Northwest, where research on CSA was unavailable. A significant number of the studies were community-based (*n* = 12). Nine studies were conducted in clinical settings, five in school settings, and only one study explored child sex offenders in prison (Aborisade et al., 2018). Twenty-five of the studies collected data from children and adolescents, particularly girls.

**Table 2.** Summary of the Included Study.

ID	Authors and Years	Design and Method of Data Collection	Setting and Geopolitical Zone	Participants	Results and Conclusion	Limitation
1.	Obisesan et al. (1999)	Quantitative Cross-sectional study Questionnaire Multistage sampling	Seven Local Government Areas Oyo State South West Nigeria	4,000 (2,000 men and 2,000 women) *Occupation*: Not mentioned *Age*: Adult	*Prevalence*: 2.1%, 5% had sex between ages 6 and 10. *Contributing factors*: living in boarding houses. Boys are more sexually abused than girls (2.4%) *Conclusion*: There is a great need for more studies on child sexual abuse (CSA) in Nigeria	Memory recall and response bias Limited reliability and validity
2.	Ogunyemi (2000)	Mixed method Interview, Focused group Questionnaire Random sampling techniques	Ijebu Ode Community Ogun state, South West Nigeria	958 participants *Occupation*: Market leaders, Religion leaders, School Principal, Occupational leaders *Aged*: Adult	*Prevalence*—Girls-38%; Boys-28% *Leading cause of sexual initiation*: Rape/date rape and child prostitution were frowned at, but gender-role stereotyping still exists. *Reason for first sexual experience among respondents*: date rape, boyfriend/girlfriend relationships, and pornography. *Conclusion*: Gender stereotypes and social stigma affect CSA disclosure. Secrecy around sex creates barriers to disclosure for girls. Empowering girls to share their experiences is important.	Response and recall bias.
3.	Ajuwon et al. (2001)	Quantitative Questionnaire Random Sampling technique	Ibadan South-West Nigeria	1,025 *Occupation*: Adolescent students and Apprentices *Age*: 15–19 years	*Prevalence*—68%–70% *Students*: 68% of females and 42% of male *Apprentices*: 70% of females and 40% had experienced at least one coercive behavior. Over 50% of girls have collected money or gifts for sex. *Perpetrator*: Boyfriends and adults male. *Four common types of sexual coercion experienced*: unwanted hand holding/unwanted sexual touch, verbal threat, unwanted kiss, and breast touch.	Limited reliability
4.	Audu et al. (2009)	Quantitative Questionnaire Simple random sampling	Maiduguri Nigeria NorthEast	316 girls *Occupation*: Sales girls *Age*: 8–19	*Prevalence*: 77.7% *Perpetrators*: Customers. Girls under 12 years not in formal education, working more than 8 hr/day, or having two jobs are at higher risk of sexual assault.	Limited reliability
5.	Bankole et al. (2008)	Quantitative Questionnaire Random Sampling	University College Hospital, Ibadan Southwest.	175 participants *Occupation*: Dentists *Age*: Adult	39.4% of the dentists suspected child abuse in one or more of their young patients; however, only 6.9% had actually reported. The possible effects on the child, uncertainty about the diagnosis, and fear of litigation	Small SampleLimited generalization
6.	Ikechebelu et al. (2008)	Quantitative Descriptive survey Questionnaire Convenient Sample	Two urban settlements Anambra State South East	186 girls *Occupation*: Street hawkers *Age*: 7–16	*Prevalence*: 7 in 10 female (69.9%) 17.2% had actual penetration. 75% did not disclose, 25% disclosed to family members and friends and only one case was reported to police.	Small sample Limited generalization
7	Oteh et al. (2009)	Qualitative Interview Purposive sampling	Ezza community Ebonyi state. South East	60 participants Fifty children (50) and ten (10) parents	Majority of the parent claimed they subjected their children to abuse because of economic burden. *Result*: Exploitation prevents child education (35%), reduces their future capacity (40%), and reduces their economic contribution (20%).	Limited generalization
8.	Aderinto (2010)	Qualitative Focus Group Discussions Interviews Convenient sampling	Ibadan, Southwest Nigeria	Adolescents girls *Age*: 18–20 years Communities and religious leaders *Age*: Adults	*Causes*: Child labor is a common cause of CSA. *Disclosure*: Victims often confide in friends and family, rarely reporting abuse to the police. In some cases, perpetrators marry or disappear. *Suggestion*: Government should outlaw early marriages, children dropping out of schools, street trading, and child labor.	Limited generality
9.	Abdulkadir et al. (2011)	Quantitative Retrospective study of cases	General Out-patient Department, General hospital Minna. North Central Nigeria.	32 cases of penile penetration	Most cases reported are children under 17, with 75% aged 6–15. Only two boys out of 32 cases were reported. *Form of abuse reported*: vaginal penile and anal penetration. Only four cases presented with 24 hr, 21 after 72 hr. *Conclusion*: Healthcare providers need to build their capacity to manage CSA and its long-term consequences	Non-in-depth response
10.	Ige and Fawole (2011)	Quantitative Study Questionnaire	Idikan Ibadan Southwest Nigeria	387 parents *Occupation*: Petty Traders And Artisans *Age*: Adult	All have good knowledge of CSA. >90% discuss with children stranger danger. 47% felt their children could not be abused, and over a quarter (27.1%) often left their children unsupervised. *Common approaches to identifying CSA*: genital or anal injury checks and signs of abnormal sexual interest in their children.	Non-in-depth response
11	Adeleke et al. (2012)	Quantitative study Retrospective study descriptive statistics and Chi squire test.	State hospital, Asubiaro, Osogbo, Nigeria South West	Hospital records of victims	Most of the victims were under 18 years old and single. Around 81% of those under 18 were abused during the day. A majority (79.6%) knew their assailant. About 40% of the victims presented within 24 hr of sexual abuse, but none had postexposure prophylaxis. *Conclusion*: Sexual assault among women is an important health problem in this environment. There is a need for hospital-based management protocol.	Non-in-depth response
12.	Ige and Fawole (2012)	Quantitative Retrospective cross-sectional study. (June 2008–May 2009)	University College Hospital Ibadan Southwest Nigeria	Age: 3–17 years	Cases were reported between 1 hr and 30 days. About three-quarters of patients in this study had investigations for STI (Sexually Transmitted Infections). Only 34% received antibiotics, with few getting counseling or contraceptives. *Conclusion*: CSA victims’ healthcare needs in Nigeria are underserved.	Non-in-depth response
13.	Olatunya et al. (2013)	Qualitative Retrospective, descriptive study	Ekiti State University Teaching Hospital South West	28 cases of CSA *Occupation*: Not mentioned *Age*: 4–17 years	*Perpetrator*: Adult male. *Management*: 60.7% were screened for hepatitis B and C and HIV. None was given prophylaxis against viral hepatitis B and C. The police were involved in 60.7% of cases, but there was no prosecution.	Clinical and small sample.
14.	Aborisade and Vaughan (2014)	Qualitative study Interview Convenient Sampling	Tai Solarin University of Education South West	27 Participants 23 rape victims and four key informants	*Prevalence*: Over 50% experienced stranger and gang rape. Only 3 out of 23 received family support. Over 60% of the 23 victims faced secondary victimization from their close ones. Only two victims fully adjusted to the situation, while none of them sought legal action. Only one victim received comprehensive medical and psychological care. *Consequence*: psychological problems (suicide attempts, depression, post-traumatic stress disorder, emotional (fear and anxiety), and behavioral difficulties.	Response bias
15	Akinlusi et al. (2014)	Quantitative Retrospective study Epi-info 3.5 statistical software	Lagos State University Teaching Hospital, Ikeja, between January 2008 and December 2012. South West	304 case notes reviewed	Out of 304 sexual assault cases, 287 had sufficient information. The majority of victims were under 19, knew attackers, and assault happened in neighbors’ homes. Over 60% presented after 24 hr, and threats/violence were common. Adolescents need protection skills, and survivors delay care. *Conclusion*: Early interventions and comprehensive care of survivors with standardized protocols are recommended.	Non-in-depth response
16	Badejoko et al. (2014)	Quantitative study Retrospective analysis- 5 years (2007–2011)	Obafemi Awolowo University Teaching Hospitals, Ile-Ife South West	Hospital records of 76 SA survivors managed	Perpetrators were known by victims, attacks often happened in the attacker’s home, and weapons were used in 29.6% of the cases. About 28.2% of survivors sustained injuries. Only 12.7% of victims came to follow-up visits. *Conclusion*: Personnel training, protocol development, provision of rape kits, free treatment, and public enlightenment on preventive strategies are recommended.	Non-in-depth response
17.	Chinawa et al. (2014)	Quantitative, Questionnaire Systemic Sampling	Private Secondary school setting Enugu Metropolis South East Nigeria	372 Teenagers, 192 females and 180 males.	*Prevalence* -10.2% 81.6% of which were females. 42.1% experienced unwanted sexual intercourse. 44.8 % were emotional, while 16.8 were physically abused. *Conclusion* To combat medical malpractice, we need to raise awareness and implement zero-tolerance laws.	Limited reliability and validity
18	Ashimi et al. (2015)	Quantitative: Prospective longitudinal study -2 years	Gynecological Emergency Unit of a Tertiary Health Facility Northwest	24 case notes of children under 16	*Prevalence*: 91.7% of case notes belong to children under 16. 45.8% (11/24) had no formal education, while 33.3% (8/24) hawked homemade drinks and snacks. *Conclusion*: The assailants were known in 83.3% (20/24) of the cases. The perpetrators are known; they were neighbors, customers and family members.	Non-in-depth response
19.	Elias et al. (2015)	Quantitative study Questionnaires Simple random sampling	Three secondary schools, Enugu and Ebonyi state South east, Nigeria.	506 Participants *Age*: 10–24 years	*Prevalence*: Overall prevalence and one-time prevalence are 40% and 11.5%, respectively, and almost half had lost count of pattern. Females were more vulnerable four times. It is also noted that 1 in 4 girls (25%) are sexually abused by the age of 18. *Commonest form of abuse reported*: Pornographic pictures, films, videotapes, or magazines. Perpetrators exposing genitals and masturbating and coerced into full sexual intercourse; vaginal or anal penetration.	Limited Generalization
20	Manyike et al. (2015)	Quantitative Cross-sectional study Simple random Sampling	Three secondary schools in Enugu and Ebonyi state. South East Nigeria.	506 adolescents *Occupation*: Secondary school students. *Age: 10–24 years*	80% were educated by parents, majority by mother only (46.2%) and both parents (45.2%). 72.1% were not informed that family members and friends can sexually abuse them. 73.8% were not informed to report to adults if it happened to them. *Conclusion*: Adolescents educated by parents were 1.23 times less likely to be abused compared to non-educated adolescents.	Non-in-depth response
21	Kunnuji and Esiet (2015)	Quantitative Questionnaire Convenient sample	Iwaya Community, Lagos State, South West Nigeria	480 Adolescents girls *Occupation*: Out-of-school students	Prevalent rate- 18% experienced coerced sex Statutory rape: 45% An association between age and experience of statutory rape.	Limited Generalization
22	Nwolisa et al. (2016)	Qualitative: Case review Retrospective analysis of medical records	Mirabel Center, Ikeja Lagos State South West Nigeria	153 cases of sexual assault *Age*: under 18 victim *Occupation*: Not mentioned	148 out of 153 patients were victims of rape-96.7% There were 147 (99.3%) females and 1(0.7%) male. Sixty-one (41.2%) knew their assailant(s), while 85(57.4%) did not know. While 101(68.2%) victims had achieved menarche, 47(31.8%) had not. In the rape of 67.6% of victims, no weapons were used while in 27% a weapon of some sort was used.	Non-in-depth response; Limited Generalization
23	Aborisade et al. (2018)	Qualitative Interview Purposive sampling	Ikoyi Prison Kirikiri Medium and Maximum Prisons Ikoyi, Lagos state South West Nigeria	29 perpetrators of child under 15 currently in prison *Occupation*: Not mentioned *Age*: Adult	Majority of their victims are under 12 years old. Childhood sexual abusive experience is an indicator of abusive behavior in adulthood. *Excuses*: (58.62%) stated, “I did not know what I was doing.” 13.79%- state of drunkenness 10.35%- ignorance of the law of child age to give consent for sex. 3.45%- attributed it spiritual machinations. 19 offenders express remorse for their actions and acknowledge the harm caused to their victims. Eight offenders only feel remorse due to the impact of imprisonment on their families. 2 offenders claim innocence.	Recall and response bias
24	Nlewem and Amodu (2017)	Quantitative study Cross-sectional study Questionnaire	Three secondary school Aba zone, Abia State Nigeria, South Eastern Nigeria	350 Student Female adolescent only *Occupation*: Students *Age: 13–17* years	Prevalence 42.5% rate among ages 13–15; 48.5% rate among 16–17 years. Female adolescents living with parents are two times less likely to be sexually abused, and female adolescents with separated or divorced parents are six times likely to be abused.	Non-in-depth narratives
25	David et al. (2018)	Quantitative Questionnaire Multistage sampling technique	Mushin Community Lagos South West Nigeria	398 adolescents *Occupation*: Not specified *Age*: 10–19 years	*The prevalence-* 25.7% (Penetrative abuse- 7.5%, Forced sex- 46.2%) *Type of sexual abuse*: Kissing, touching private parts, flashing, showing pornographic magazines/films, took pictures of me naked, sexual intercourse *Disclosure*: 61% did not, 34.4% disclosed *Reason for Non-Disclosure*: Social shame and guilt, and nothing would come of my telling.	Reliability and validity problems
26	Okeafor et al. (2018)	Quantitative Case-control study Systematic sampling	University of Port-Harcourt Teaching Hospital in Rivers State, South-South	304 (Case-152, Control—152) *Occupation*: Non-specified *Age*: 18–60	*Prevalence*: 21.4% Exposure to CSA is associated with mental illness in adulthood (adjusted odds ratio = 3.11, 95% CI [1.67, 5.82]) and family functionality.	Reliability and validity problems
27.	Olatosi et al. (2018)	Quantitative Questionnaire Convenient sampling	Lagos University Teaching Hospital, Lagos. Southwest	179 respondents *Occupation*: Dentist *Age*: Adult	Physical, sexual, and emotional abuse and neglect were majorly identified as bruises behind the ears, 162 (90.5%); oral warts, 114 (63.7%); poor self-esteem, 158 (88.3%) and untreated rampant caries, 137 (76.5%), respectively. Only 12 (14.1%) of those who observed suspected cases reported to the social service.	Limited generability
28.	Oyekola and Agunbiade (2018)	Mixed method Questionnaires Interview	Ile-Ife and Modakeke Southwest Nigeria	443 Adolescents *Age*: 9–20. 10 teachers	*Prevalence: 59.8%* *Management*: Counseling, followed by informing parents or friends. Majority prefer to keep to self. Parents’ negative involvement in their children’s sexual issues suggests that child education, security, and strict punishments could help address the problem.	Recall and response bias
29	Sodipo et al. (2018)	Quantitative: Retrospective Audit	Mirabel Center, Lagos State University Teaching Hospital Lagos State Southwest	2,160 cases of rape Three years case review	*Survivors*: female 97.7%, Male: 2.3% *Perpetrator*: The majority of the perpetrators were known to the survivors with 10.3% being family members. *Common form of abuse*: Defilement (71.6%) Rape (20.3%). The majority of the referrals to the center were from the police (76.7%), while self-referrals made up 8% of referrals.	Secondary data Reliability and validity problem
30.	Opekitan et al. (2019)	Quantitative cross-sectional Questionnaire Purposive	Twenty selected health facilities in Ogun state. Southwest Nigeria	86 respondents Occupation: Health workers	A large percentage of health workers ( more than half) were unaware of any social infrastructure or hospital protocol for child abuse reporting. Many health workers lack awareness of social infrastructure or hospital protocol for reporting child abuse. Deliberate training is needed to help victims of child abuse.	Small sample Limited Generalization
31	Uvere and Ajuwon (2021)	Quantitative Cross-sectional study Questionnaire Descriptive statistics, chi-square and logistic regression	Selected market in Ibadan. SouthWest	Female Adolescent hawkers (FAHs) 410 participants	69% of young female hawkers faced sexual abuse within 3 months prior to the study. Male customers, traders, and peers perpetrated most of the abuse. Shockingly, 67.5% of victims didn’t seek help. To address this issue, interventions such as age-appropriate sexuality education and life-building skills should be targeted toward FAHs. *Conclusion*: Advocacy is also recommended for caregivers and market stakeholders.	Recall and response bias

*Note.* This systematic review includes 31 articles, and the table presents a summary of the key findings from these articles.

Only eight (Aborisade & Vaughan, 2014; Aderinto 2010; Ebuenyi et al., 2018; Ige & Fawole 2011; Ogunyemi, 2000; Olatosi et al., 2018; Opekitan et al., 2019; Oyekola & Agunbiade, 2018) study explored the experiences of parents, community leaders, religious leaders, HCPs, teachers, and offenders, and none interviewed policy makers. Purposive and convenience sampling techniques were commonly used. Sample sizes ranged from 23 to 4,000 participants (Obisesan et al., 1999; Ashimi et al., 2015).

### Data Collection

Data were collected using questionnaires (*n* = 18). Most of these questionnaires were self-developed and considered the social lifestyle and factors that could predispose their participants to be sexually abused in childhood. Also, the age of onset was commonly elicited across the questionnaires, as was the relationship between the victims and the perpetrators. Some studies used only 6-item questionnaires (Adeleke et al., 2012), while others used as many as 30 (Ajuwon et al., 2001). Other methods of data collection include in-depth qualitative interviews (*n* = 5), and two studies used a mixed approach to data collection, which included questionnaires, in-depth individual interviews, and focus groups (Ogunyemi, 2000; Oyekola & Agunbiade, 2018).

### The Prevalence and Patterns of CSA

A wide variation in the prevalence of CSA in Nigeria was reported, ranging between 2.1% and 77.7% (Audu et al., 2009; Obisesan et al., 1999). Of the 31 included studies, 12 reported a higher prevalence of CSA in females, for instance (Audu et al., 2009; Ogunyemi, 2000). Only one study, however, showed that more boys (47, 2.4%) than girls (34, 1.7%) experienced CSA (Obisesan et al., 1999). To understand the disparity in prevalence, we looked at the acts that constitute CSA in each study. According to Obisesan et al. (1999), childhood is defined as the age between 6 and 10 years, which is different from the generally accepted definition of any child below the age of 18. Additionally, the acts that constitute CSA in this study were limited to penetrative vaginal intercourse. Participants were only asked if they were forced to have sex as a child, neglecting other non-penetrative sexual activities that may have been experienced by female participants. This may explain why the prevalence of CSA was lower in this study compared to others that assessed a wider range of sexual activities.

A variation in prevalence was observed depending on the group; for example, the prevalence of CSA was reported to be 70% among apprentices (Ajuwon et al., 2001), 69.9% among girls selling goods on the street (Ikechebelu et al., 2008), 10%–68% among teenagers attending secondary schools (Envuladu et al., 2013; Manyike et al., 2015), and 35% in out-of-school children (Kunnuji & Esiet, 2015). The most common age of first exposure was reported to be 12, and those under the age of 18 years were more likely to be victimized again within the next year (Kunnuji & Esiet, 2015).

### Forms of CSA Reported

Common forms of CSA reported were unwanted kissing, hugging, inappropriate touch to breasts and genitals, verbal threats, abuse, and rape (Abdulkadir et al., 2011; Ajuwon et al., 2001; Ikechebelu et al., 2008). In addition, teenagers were forced to record themselves naked, look at pornographic pictures, films, videotapes, or magazines, watch perpetrators exposing genitals and masturbating, coerced into full sexual intercourse, or experience rape and vaginal or anal penetration (David et al., 2018; Manyike et al., 2015; Okeafor et al., 2018; Oyekola & Agunbiade, 2018). Over 50% of children that participated in this study experienced stranger and gang rape (Aborisade & Vaughan, 2014). Child marriage is not recognized as CSA in reviewed articles despite its prevalence in Nigeria. This may be due to its social and cultural acceptance, along with inconsistencies in the Nigerian constitution regarding the age of marriage consent.

### Perpetrators and Their Strategies

All the studies found that perpetrators were usually men, mostly known to the victims (Adeleke et al., 2012), either as relatives, friends, customers, or neighbors. Only 20% of the respondents were victims of stranger or gang rape. In a study by Badejoko et al. (2014), it was found that neighbors were the most commonly identified perpetrators. Eight studies found that perpetrators used different forms of enticement, such as money, gifts or food, alluring promises, shelter or accommodation to lure adolescents (for instance, Aborisade et al., 2018; Adeleke et al., 2012; Ajuwon et al., 2001). Other means of subjugation include verbal threats and the actual use of force. Badejoko et al. reported in 2014 that in 29.6% of cases, perpetrators were violent and used weapons to threaten the victims. The most commonly used weapons for threatening are guns and knives, followed by bricks, broken bottles, machetes, and shaving blades (Badejoko et al., 2014; Nwolisa et al., 2016).

### Risk Areas/Locations

Common places reported for CSA by perpetrators known or related to the child were during times of being home alone with the child, watching TV with the child, or when sending the child on an errand after gaining the parent’s trust (Aborisade et al., 2018; Adeleke et al., 2012; Akinlusi et al., 2014). Attacks by known assailants frequently occur in the attacker’s residence (Badejoko et al., 2014). The most common places for unknown assailants to coerce the child into sexual activity were friend’s homes, familiar neighborhoods, and during organized activities, such as parties (Aborisade et al., 2018). Evidence from this review showed that a significant number of children are sexually abused when hawking different forms of goods (Ashimi, Amole & Ugwa 2015). Assailants’ offices or business premises were the location for 40.6% of the cases, while 25.0% occurred in residential homes and 21.8% inside motor vehicles (Ikechebelu et al., 2008). Other locations included market stalls and mechanic workshops.

### Victim’s Awareness of Whether CSA Can Lead to Pregnancy

One particular study by Ikechebelu et al. (2008) considered children respondents’ awareness of the risks associated with unprotected sex. 56.9% and 45.7% were unaware that such coercion could lead to unwanted pregnancy and sexually transmitted infections, respectively. It is important to educate young people about the risks of sexual abuse and to provide them with information about the support they can receive from their social network or protective agencies.

### Help-Seeking Behavior or Intention to Disclose the Experience

A significant percentage (31.5%) of girls who participated in this study claimed they bought self-prescribed medication at a pharmacy after experiencing abuse. Most of the victims preferred to keep the experience to themselves, and few victims discussed their experiences with friends and family members. It is important to know that 75% of the participants chose not to disclose their experience, while 25% felt comfortable enough to share with family and friends. Only one case was reported to the police. This highlights the importance of creating a safe and supportive environment for individuals to feel comfortable coming forward with their experiences (Ikechebelu et al., 2008).

### Factors Associated with CSA in Nigeria

Twelve studies explored the causes of CSA, for example (Aderinto, 2010; Fawole et al., 2002; Nlewem & Amodu, 2017; Obisesan et al., 1999). One study by Nlewem and Amodu (2017), found that female adolescents living with their parents were less likely to be sexually abused than female adolescents with separated or divorced parents. Other factors that increased the chances of CSA included basic deprivation and living arrangements (Kunnuji & Esiet, 2015). Several factors have been identified that increase the risk of child labor and exploitation practices, such as hawking and living separately from parents. Also, factors include being of a younger age range, specifically between 10 and 15 years old, consuming alcohol, having a disability, and experiencing labor and exploitation practices (Aderinto, 2010; Envuladu et al., 2013; Fawole et al., 2002). Ikechebelu et al. (2008), however, identified no significant relationships between the incidence of adolescent sexual abuse and socioeconomic class or age.

Additionally, research exploring teenagers’ perspectives has shown that the most common causes of CSA are poverty (52%) and cultural and religious practices (28%). Most children that participated in these studies in Nigeria suggested that their parents’ low socioeconomic status and inability to meet their financial needs subjected them to exploitative practices such as hawking, street begging, and seeking employment as house maids, which, although appeared to solve their financial precarity, also indirectly exposed them to CSA (Oteh et al., 2009; Oyekola & Agunbiade, 2018). Beyond this, gender discrimination and the relative social invisibility of young females alongside prevailing societal norms that are supportive of sexual violence were identified as other causes of CSA in Nigeria (Ajuwon et al., 2001; Manyike et al., 2015; Olatosi et al., 2018). The least-reported factors were physical appearance and lack of sex education (Oyekola & Agunbiade, 2018). Two studies identified protective factors, such as the active involvement of parents and teachers in terms of early sexual education and raising awareness of CSA to girls (Manyike et al., 2015; Oyekola & Agunbiade, 2018).

### Nigerian Parents’ and Victims’ Knowledge, Perceptions, and Attitudes Regarding CSA

Six studies explored participants’ knowledge, perceptions, and attitudes, with emphasis on their sociocultural perspective and how this frames their standpoint regarding CSA (Aborisade & Vaughan, 2014; Aderinto, 2010; Ige & Fawole, 2011; Ogunyemi, 2000; Oyekola & Agunbiade, 2018; Ebuenyi et al., 2018). Ige and Fawole (2011) revealed that the majority (78.3%) of parent respondents had previous knowledge, as well as having heard about an incident involving their child or another child. Of these, merely 18.8% defined CSA as sexual intercourse with a child, either forcefully or consensually. Unfortunately, this study did not report how the remaining 80% of respondents defined CSA. The majority (84.2%) of the respondents agreed that CSA is common in their community; however, only (34.6%) agreed that CSA could have a serious health impact on victims. Only 2.1% of parents disclosed their children had experienced CSA, and more than 90% of respondents claimed they discussed “stranger-danger” with their children. Despite parental awareness, almost half of the respondents claimed their children could not be sexually abused, yet the authors subsequently note that over a quarter reported leaving their children unsupervised. Eighty percent of respondents condemned CSA acts such as rape, date rape, gang rape, child prostitution, and incest; however, evidence of gender-role stereotyping exists (Ogunyemi, 2000), and as a result, female gender rights are seen as an appendage to males, due to boy-child preferences in Nigeria. Ige and Fawole (2011) explored parents’ perspectives of practices that could contribute to CSA and found that respondents agreed they should sell their children to whoever can feed or properly educate them, especially in instances of extreme financial poverty. Aderinto (2010) reported that victims of sexual abuse are frequently forced to marry their perpetrators, especially when the sexual abuse results in unplanned pregnancy. Sexually abused children who participated in this study claimed they did not disclose that they had been a victims of CSA to their parents. Instead, 75% preferred to discuss their abuse with a friend. Others (Oyekola & Agunbiade, 2018), however, found that some children still reported their experience of CSA with family members and friends. The number of parents who disclosed their child’s experience to the police, community leaders, or an HCP was negligible. This may be due to the current trend in Nigeria, where victims are subjected to secondary victimization by their parents, medical personnel, families, neighbors, and others (Aborisade & Vaughan, 2014; Ebuenyi et al., 2018). The reviewed studies found that more efforts are required within the school system and at household and societal levels to curb, manage, and reduce CSA. Victims must be referred for counseling, and perpetrators must be severely punished (Ebuenyi et al., 2018). These studies identified the strengths and gaps in parents’ knowledge, perceptions, and practices of CSA in Nigeria. A significant number of parents accepted CSA as a common occurrence in their community and disagreed with common CSA myths stated in the questionnaire by the authors (Ige & Fawole, 2011). These myths include the belief that CSA is only severe when it involves intercourse, that homosexual abuse is more serious than heterosexual abuse, that female adults cannot sexually abuse children, and that only wayward or troubled children are at risk of being sexually abused. This parent’s knowledge of CSA and perception of risk can improve communication with their child and warn their children about stranger danger; unfortunately, most of the assailants are known and often trusted by parents and victims. However, despite being aware of the potential risks, it was felt by researchers (Ige & Fawole, 2011) that parents did not take tangible actions to safeguard their children.

### The Impact of Sexual Abuse Among Adolescents in Nigeria

Among the 31 papers examined, only two studies (Aborisade & Vaughan, 2014; Oteh et al., 2009) predominantly focused on the immediate and long-term sequelae of abuse on adolescents, society, and the nation at large. These studies identified psychological consequences such as flashbacks, sleep disorders, guilt and self-blame, feelings of powerlessness, distrust, and anger. Other psychological consequences are suicide attempts, depression, post-traumatic stress disorder, and emotional (fear and anxiety) and behavioral difficulties (Aborisade & Vaughan, 2014). Sixty-eight percent of young people explained that they were also victimized by their parents, medical personnel, families, friends, and neighbors. Participants claimed their experiences with CSA left them traumatized. Child respondents in this study affirmed that such traumatic experiences damaged their educational careers, reduced the country’s future workforce, and impaired their future contribution to economic development, and thereby, CSA created long-standing negative impacts on their nation (Oteh et al., 2009). Consequently, the effects of CSA have a far-reaching and devastating impact on the individuals and the nation as a whole.

### HCPs’ Knowledge, Perceptions, and Attitudes Regarding CSA

Three studies (Aborisade & Vaughan, 2014; Olatosi et al., 2018; Opekitan et al., 2019) focused on HCPs and assessed their knowledge, perceptions, and attitudes about CSA. Olatosi et al. (2018) reported limited knowledge of dentist’s knowledge of indicators of child abuse and a lack of a clear structure for referring victims to essential services. Dentists are in a unique position to identify physical indicators such as unexplained injuries, bruising, or trauma to the mouth, face, or head since they routinely examine many children. Despite being one of the specialists who attends to more children and is well placed to intervene, 46.5% of respondents do not evaluate suspected cases of abuse and neglect. When the dentists were asked how they responded to suspected cases of abuse, 65.4% reported a lack of knowledge about referral procedures, and 57.5% were afraid of the consequences for the child. Findings from this study are consistent with research by Opekitan et al. (2019), which assessed HCPs’ level of awareness of the social and legal supports available for victims. Disturbingly, an overwhelming proportion of respondents in this study agreed they are unaware of the available social resources for victims. Additionally, the study shows that more than half of HCPs that participated in this study lacked adequate knowledge of referral procedures and were concerned about confidentiality issues, a predominant barrier to reporting suspicious cases of child abuse (Olatosi et al., 2018). Despite theoretical knowledge, clinical inefficiency exists, which demonstrates knowledge gaps among HCPs in recognizing and responding to victims of sexual abuse, culminating in professional non-enquiry attitudes. On the other hand, research by Aborisade and Vaughan (2014) explored rape victims’ post-assault experiences and adjustment patterns, interviewed two medical doctors and two psychologists, and collected experts’ opinions on the victimology of rape. Respondents from this study stated that victims’ reactions and recovery largely depend on a complex combination of individual characteristics (such as personality) and external factors (such as the victim’s social support network, victim-assailant relationship, and severity of the assault). As such factors have a great impact on a victim’s psychological functioning and adjustment process, it is important to focus on a victim’s individual experiences and their unique social contexts when assessing their reactions and recovery needs. This will ensure that victims are provided with appropriate and tailored support.

### Identification, Prevention, and Management of CSA Cases in Nigeria

This review identifies studies that discuss the practice of identifying CSA victims in Nigeria. Research by Oyekola and Agunbiade (2018) explored teachers’ opinions and reported complexities surrounding the recognition of CSA. In Nigeria, common methods used by parents for identifying CSA involve conducting genital or anal injury checks and looking for signs of abnormal sexual interest in their children (Ige & Fawole, 2011). Symptoms that were noted were things like young people withdrawing from others, being sad or moody, displaying anxiety or seeming to be in pain, and finding difficulty in carrying out daily activities. Studies suggest that primary prevention in contemporary Nigeria is based on parental supervision and child-parent communication about sexual activity and danger from strangers and familiar people, while secondary preventive practices include reporting to the police station and hospital for medical examination (Oyekola & Agunbiade, 2018; Ige & Fawole, 2011). The study conducted by Ige and Fawole in 2011 highlighted the crucial importance of promptly reporting to the police and seeking a medical examination in suspected cases of CSA. This approach is essential for the early detection of CSA cases and increases the likelihood of victims accessing early treatment with the goal of preventing harm to potential victims and promoting community safety. Unfortunately, authors reflect that discussions around sexual abuse seldom occur, and parental supervision is neglected (Ige & Fawole, 2011). Also, this study revealed that although Nigerian parents can readily identify the immediate physical signs of CSA, they were often unable to recognize behavioral changes as potential indicators of CSA. Such unawareness about the range of symptoms associated with CSA may delay the needed response to protect the child from further abuse and seek treatment. In cases where abuse is identified, they are rarely reported as respondents believe disclosing such acts will only bring social stigma and more trauma for their child and family rather than justice (Ogunyemi, 2000). The way parents respond to CSA is crucial as it greatly influences the child’s chance to access, disclose, and receive support from legal and health professionals after experiencing abuse. Of the 31 included studies, two focused on management of CSA (Olatunya et al., 2013; Ige & Fawole, 2012). Only 50% of victims from cases reviewed were subjected to routine High Vaginal Swabbing and retroviral screening, including for hepatitis B and C, and none received HIV and viral hepatitis postexposure prophylaxis, which is necessary to prevent them from being infected with the HIV virus. Out of the 60.7% of cases reported to the police, none led to prosecution (Ige & Fawole, 2012; Olatunya et al., 2013). Antibiotics were only prescribed to 34% of the victims, with fewer prescriptions for analgesics, vitamins, counseling, and contraceptives; only three quarters of the victims were checked for sexually transmitted infections, and a negligible number of victims were referred to postexposure prophylaxis, while none received the hepatitis B vaccine (Ige & Fawole, 2012). These studies reported that cases were reported between 1 h and 30 days after occurrence of the abuse, generically managed, and not reported to the police. HCPs only come in contact with children when they are presented by the parent, guardian, or teacher, and there are no structural systems in place to report abuse; most cases go unnoticed as there is no routine screening for child abuse among young girls in the Nigerian healthcare system. Previous studies have clearly identified that parents are less likely to report the sexual abuse of their child because of the social stigma and future consequences for the child; this suggests, therefore, that only a minority of CSA victims are referred for medical intervention.

## Discussion

The purpose of this study was to systematically aggregate empirical evidence and critically review the existing body of knowledge on CSA in Nigeria. Firstly, while general research on CSA has increased over the past two decades, studies focusing directly on CSA in Nigeria are still rare. This review highlights that limited research has been conducted on CSA in Nigeria. Twenty-three of the reviewed studies were carried out using quantitative approaches to explore the prevalence experiences of CSA victims and HCPs (Adeleke et al., 2012; Ajuwon et al., 2001; Badejoko et al., 2014; Olatosi et al., 2018). Yet CSA experiences are complex and sensitive, and using a quantitative approach can decontextualize the complexity of that experience. Some of the researchers that used clinical case studies seem not to be sensitive to the context, and the possible influences of this approach on the reliability and generalization of these studies were not mentioned (Ige & Fawole, 2012; Nwolisa et al., 2016; Olatunya et al., 2013; Sodipo et al., 2018).

More than half of studies included in this review predominantly focused on the prevalence and patterns of CSA. Twelve study including (Aderinto, 2010; Fawole et al., 2002; Kunnuji & Esiet, 2015; Nlewem & Amodu, 2017) focused directly on the factors responsible, and little effort had been made to examine the current clinical patterns of management. Only two focused on the knowledge and awareness of HCPs, and none focused on the challenges faced in identifying and responding to a victim of CSA. None of these studies directly explored young people’s and adults’ perceptions about children’s status in society and their association with CSA. Therefore, it is clear that more research needs to be done to fully understand the extent of CSA in Nigeria.

According to this review, there are disparities in published statistics across the country concerning CSA, and the actual magnitude of the dilemma remains unknown. For the development of prevention strategies and policy initiatives, it is crucial to have accurate data on the magnitude of CSA (Finkelhor et al., 2016; Joleby et al., 2020). Despite growing evidence of the size of the problem, current evidence comes largely from institutionalized settings, and 18 used community-based samples. Another concern is the diverse and varied terminology employed and variation operation definition of the constructs of CSA used by researchers and research methodologies limit the extent to which comparisons can be made between studies (Mitchell et al., 2017). The current review showed definitional ambiguities, the heterogeneous nature of victims and perpetrators, and the rapidly changing nature of sexual activities have masked the true scale of this crime. This review also found that discrepancies and wide variation in the prevalence of CSA in Nigeria could be attributed to the gender of participants, that is, the number of males and females and the use of broad terminology rather than a specific definition of one of the named types of abuse. A definition of CSA in general, but most importantly, a distinction between types of sexual abuse, is obvious. Future research should develop a universal definition and language for CSA, as well as a clear distinction between types.

This review highlights that children are at a higher risk of experiencing sexual abuse due to sociocultural norms and their parents’ low socioeconomic status. However, several studies from other countries have identified potential vulnerabilities and indicators that increase the risk of child sexual exploitation and concluded that these risks apply to all children, regardless of their gender, ethnicity, cultural background, or socioeconomic status (Davies & Jones, 2013; Franklin et al., 2015; Wodarski & Johnson, 2015). This means that having a reductionist approach to theorizing the predisposing factors to CSA may affect the development and implementation of practice and policies to safeguard children and identify and respond to victims of CSA.

This review showed that CSA entails short-term and lifelong sequela for the individual, family, and society, especially if left unrecognized or untreated (Hornor, 2010; Mullers and Dowling, 2008; Warrington et al., 2017). Early trauma suffered by sexually abused children has been linked to multiple behavioral, psychiatric, and mental problems, including substance abuse, anxiety and depression, post-traumatic stress disorder, and suicide attempts (Mullers & Dowling, 2008; Okeafor et al., 2018; Warrington et al., 2017). This field requires further research, given the high prevalence of CSA in Nigeria and the severe effects it has on victims’ wellbeing, as well as the restrictive challenges professionals face in safeguarding them (Ifayomi, 2023). The harmful and sociocultural practices, taboos, and shared norms and values remain a major predictor for CSA in Nigeria and barriers to disclosing and seeking help. Despite the parental awareness, almost half of the respondents claimed their children could not be sexually abused and also left their children unsupervised. Hence, parents’ perspectives, practices, and attitudes could also contribute to CSA.

Radical services and approaches are in place for the protection and social welfare of children and the prevention of CSA (Nelson, 2016). This approach is contrary to the current situation in Nigeria, where the quality of medical and psychosocial care provided to survivors of CSA remains poorly studied and has proved to be substandard compared to the required care for victims (Akin-Odanye, 2018). There is still a substantial gap between the medical services provided to victims of CSA and their healthcare needs (Aborisade et al., 2018; Akin-Odanye, 2018; Ige and Fawole, 2012), and HCPs expressed their frustration as their efforts remained unproductive. There is need for more research on CSA in Nigeria regarding the predisposing factor, available service for victims and family, and professional practice of supporting sexually abused children in Nigeria. Summaries of critical findings and implications are included in Tables 3 and 4.

**Table 3.** Critical Findings.

The risk and vulnerability factors that increase acts of sexual violence and abuse against children include basic deprivation, extended living arrangements, younger age (child aged 10–15 years), alcohol consumption, disability, child labor, and exploitation practices, such as hawking. Prevailing societal norms cause gender discrimination and social invisibility. Active involvement of parents in sexual education and orientation of girls on Child sexual abuse (CSA) are good protective factors.
Parents can readily identify the immediate physical signs of CSA, but they are unable to recognize behavioral changes as indicators; such unawareness may delay the needed response to protect the child from further abuse and seek treatment.
Psychological consequences such as flashbacks, sleep disorders, a sense of guilt and self-blame, emotional numbness, the feeling of personal powerlessness, a sense of distrust, and anger. Victims were found to be left traumatized with unsavory memories, which tend to truncate psychosocial development. Impacts of experiencing traumatic experiences damage their educational career, reduce the country’s future workforce, and impair their future contribution to economic development.
Professionals were not confident in addressing cases and limited knowledge on knowledge of referral procedures and identifying cases.
There existing care for sexually abused children remains substandard, as there is a wide gap between the available structures of CSA management and the care the victims require.

*Note.* A summary of critical findings from current systematic review.

**Table 4.** Implications of the Review for Practice, Policy, and Research.

Practice	Low level of society awareness of Child sexual abuse (CSA), common practice and perception, and available substandard healthcare suggest professional knowledge, perception of their roles, and evaluation of care provided for sexually abused children is warranted.
Policy	The enormous magnitude of the problem and existential dimensions of CSA-related professional services demands that policymakers develop and implement all-encompassing child-oriented policies and child and family-focused practices.
	There is a need for urgent and comprehensive context-based and culturally sensitive national guidelines for healthcare professionals working with sexually abused children in Nigeria.
Research	Most research conducted in Nigeria on CSA has been quantitative; CSA experiences are not only complex and sensitive but subjective in nature, and using a quantitative approach decontextualizes the complexity of that experience. There is a need to conduct rigorous studies that explore how Nigerian define CSA and the personal meaning the victims of this crime attributed to it and to examine their attitudes toward CSA.
	There is a need to conduct phenomenological qualitative studies to understand society’s perceptions, attitudes, and victims’ experiences of CSA. Such empirical evidence can then be utilized to develop culturally specific and context-oriented instrument quantitative studies.
	There is an urgent need to explore those factors undermining professional practice of supporting sexually abused children and develop context-based national guidelines.

*Note.* Highlights of important implications for practice, policy, and research.

## Strengths and Limitations

This review examines the prevalence of CSA in different samples and highlights the magnitude of CSA in Nigeria and the types of CSA that are most commonly experienced in the Nigerian context. In addition, the study examined the factors influencing children’s vulnerability to sexual abuse in Nigeria. Empirical evidence reveals patterns of CSA and society’s perceptions and awareness of CSA and its devastating consequences for victims, their families, and wider society. Such evidence is necessary to develop a framework for context-oriented preventative programs in Nigeria. Additionally, these results increase our understanding of available services and the gaps in literature for further research. It provides implications for public health policies and interventions and calls for the need to strengthen legal frameworks and improve the system of reporting and responding to cases of CSA. Finally, it emphasizes the need for further research on CSA in Nigeria. The critical appraisal of these studies also helped to assess the quality and quantity of studies conducted in Nigeria on this topic. A broad perspective has been offered on empirical research in CSA in Nigeria because the review has not been limited to any one research design or methodology (meaning all designs, either quantitative, qualitative, or mixed method approach studies, are included). The review is limited by the fact that we only included studies published between 1999 and 2022 in peer-reviewed journals and published in English. It is possible that some studies, which were published in non-indexed journals or non-published for some reasons, such as dissertation or thesis, are not identified and included. In selecting the studies for inclusion in the review, we ensured only studies representing independent samples and only those that explored CSA were included; others that explored other forms of child abuse and exploitation were excluded, therefore may impact the overall findings and may present an underestimate of the prevalence of CSA. Also, this review was conducted to gather information about the Nigerian context, and so the data is specifically related to the West African context. Please take into account these limitations when considering the results of the review.

We considered the diverse characteristics of the studies included in this analysis, particularly the participant demographics, topics explored, and context. Studies explored a wide range of CSA areas, including prevalence and contributing factors, parents’ perceptions of CSA, management of CSA, and support for CSA victims. The studies included in this review were conducted across Nigeria’s six geopolitical zones, although most are published from a particular zone, the Southwest. Aggregating evidence from all zones and cultures across the country helped the researchers explore different perspectives and cultures, identify the uniqueness among people from diverse backgrounds, and understand how these factors influence their behaviors, attitudes, and values. We believe this is critical for developing more effective and culturally sensitive interventions, policies, and services to meet the needs of different victims and their families. On the other hand, the lack of evidence on CSA from other geopolitical zones has serious ethical consequences for our review findings and limits generalisability, especially for victims in rural areas.

Lastly, comparing the results from this systematic review with Okunlola et al. (2020), a systematic review of 20 articles published between 2005 and 2016 on CSA in Nigeria helps to highlight the progress made in the field and areas that still warrant attention. In 2020, Okunola and colleagues made observation that most of the available studies were clinically reported cases of CSA; however, this review shows that effort has been made by researchers to start conducting community-based research, exploring the victims’ experience and society’s perspectives and practices around CSA, including perpetrator opinions and perceptions. However, many gaps in the research have not been examined. There is a need to exclusively examine the current services available to victims of CSA in Nigeria. Additionally, there remains a substantial lack of research that focuses on the challenges facing HCPs in supporting CSA victims in Nigeria. By understanding this, a more context-oriented approach, procedure, and policy that caters to the needs of victims, families, and professionals can be developed.

## Implication for Further Research, Practice, and Policy

Our review revealed that there are critical implications for further research in terms of research methodological approaches, aspects of the CSA subject explored, research samples, and implications for practice. Most research conducted in Nigeria on CSA has been quantitative. There is a need to conduct rigorous studies that explore how Nigerians define CSA, and the personal meaning the victims of this crime attributed to it and to examine their attitudes toward CSA. There is a need to conduct qualitative studies to understand societal perceptions, attitudes, and victim experiences of CSA. Such empirical evidence could then be utilized to develop culturally specific and context-oriented quantitative studies. Similarly, no empirical work has been conducted to explore how HCPs can help prevent CSA, raise awareness, and help victims emotionally and socially. Since disclosure is essential in initiating medical, psychosocial, and legal intervention for children who have been sexually abused, there is a need to explore the disclosure process, as well as barriers and facilitators from the perspectives of victim family and professionals. Most research conducted in Nigeria has not thoroughly explored the issues regarding the prevention of CSA. Lastly, since the available healthcare services for sexually abused children in Nigeria are still developing and, therefore, sometimes ineffective compared to the comprehensive care required by these victims, there is need to explore the way HCPs perceive and understand their roles and the associated issues and challenges undermining their effort from their own point of view is necessary. It is important to emphasize the use of biopsychosocial models of intervention instead of the ineffective biological model when managing cases of CSA in Nigeria. The review has shed light on certain policy gaps, emphasizing the importance of the government taking proactive measures to address CSA in Nigeria. To achieve this, the government should prioritize sponsoring public awareness campaigns and implementing a child-friendly safeguarding process to effectively prevent, identify, and provide high-quality support for victims. Additionally, there is a need for stricter, comprehensive policies to deter perpetrators and implement a strategy that aims to bring more offenders to justice and reduce reoffending. It is also crucial to establish and enforce more stringent and comprehensive policies aimed at deterring potential perpetrators of crime. Furthermore, there is a critical need to develop a comprehensive strategy that focuses on increasing the prosecution of offenders while also addressing the root causes of criminal behavior to reduce the likelihood of reoffending.

## Conclusion

Despite receiving increasing attention, CSA remains a severely and extremely detrimental epidemic. The magnitude of CSA in Nigeria remains unknown, with disparities in published statistics across the country. The sociocultural norms, including traditional practices and the low socioeconomic level of parents, increased the child’s vulnerability to sexual abuse and prevented them from disclosing the abuse. By taking proactive measures, parents can effectively reduce the risks of CSA and create a safer environment for their children. The quality of medical and psychosocial care provided to survivors of CSA remains poorly studied and has proven to be substandard compared to the required healthcare for victims. Even though the detrimental impact of CSA on victims is getting more attention around the world, professionals in Nigeria struggle to develop effective practices, services, and policies. Therefore, it is recommended to define CSA legally, develop a consensual conceptual framework, and increase awareness of CSA. The process of reporting and responding to children who have been sexually abused in Nigeria must also be improved.
